# Risk factors for cholesterol polyp formation in the gallbladder are closely related to lipid metabolism

**DOI:** 10.1186/s12944-021-01452-6

**Published:** 2021-03-22

**Authors:** Zhihao Yu, Changlin Yang, Xuesong Bai, Guibin Yao, Xia Qian, Wei Gao, Yue Huang, Xiaodong Tian, Shi Cheng, Yamin Zheng

**Affiliations:** 1 Department of General Surgery, Xuanwu Hospital, The First Clinical Medical College, Capital Medical University, No.45 Changchun Street, Beijing, China; 2 Vascular Surgery Department, Xuanwu Hospital, The First Clinical Medical College, Capital Medical University, Beijing, China; 3 Department of General Surgery, Dongzhimen Hospital, Beijing University of Chinese Medicine, Beijing, China; 4 Division of Gastroenterology, Hepatology and Nutrition, Department of Medicine, University of Florida, Gainesville, Florida USA; 5 Pathology Department, Xuanwu Hospital, The First Clinical Medical College, Capital Medical University, Beijing, China; 6 Information Center of Xuanwu Hospital, Xuanwu Hospital, The First Clinical Medical College, Capital Medical University, Beijing, China; 7 Department of General Surgery, Peking University First Hospital, Beijing, China; 8 Department of General Surgery, Beijing Tiantan Hospital, Capital Medical University, Beijing, China

**Keywords:** Risk factors, Gallbladder, Cholesterol polyps, Formation, Lipid metabolism, Multicenter, Pathology

## Abstract

**Background:**

The purpose of this study was to assess the risk factors for cholesterol polyp formation in the gallbladder.

**Methods:**

This was a multicenter retrospective study based on pathology. From January 2016 to December 2019, patients who underwent cholecystectomy and non-polyp participants confirmed by continuous ultrasound follow-ups were reviewed. Patients in the cholesterol polyp group were recruited from three high-volume centers with a diagnosis of pathologically confirmed cholesterol polyps larger than 10 mm. Population characteristics and medical data were collected within 24 h of admission before surgery. The non-polyp group included participants from the hospital physical examination center database. They had at least two ultrasound examinations with an interval longer than 180 days. Data from the final follow-up of the non-polyp group were analyzed. The risk factors for cholesterol polyp formation were analyzed by comparing the two groups.

**Results:**

A total of 4714 participants were recruited, including 376 cholesterol polyp patients and 4338 non-polyp participants. In univariate analysis, clinical risk factors for cholesterol polyps were age, male sex, higher body mass index (BMI), higher low-density lipoprotein (LDL), lower high-density lipoprotein (HDL), and higher aspartate aminotransferase (AST) and alanine aminotransferase (ALT) levels. In multivariate logistic analysis, independent risk factors were age > 50 years (odds ratio [OR] = 3.02, 95% confidence interval [CI] 2.33–3.91, *P* < 0.001], LDL > 2.89 mmol/L (OR = 1.38, 95% CI 1.08–1.78, *P* = 0.011), lower HDL (OR = 1.78 95% CI 1.32–2.44, *P* < 0.001), AST > 40 IU/L (OR = 3.55, 95% CI 2.07–6.07, *P* < 0.001), and BMI > 25 kg/m ^2^ (OR = 1.32, 95% CI 1.01–1.72, *P* = 0.037).

**Conclusions:**

Age, LDL, HDL, AST, and BMI are strong risk factors for cholesterol polyp formation. Older overweight patients with polyps, accompanied by abnormal lipid levels, are at high risk for cholesterol polyps.

## Background

Gallbladder polyps (GBP) are tumors or tumor-like protrusions on the mucous membranes of the gallbladder, with an annual incidence of 6.9%–7.6% in cholecystectomy specimens [1, 2]. Cholesterol polyps account for the majority of all GBP, ranging from 63.0%–92.0%; the remaining are non-cholesterol GBP, such as adenomatous polyps, inflammatory polyps, and adenocarcinoma [1–4]. Previous studies based on ultrasound have shown variable risk factors associated with GBP, including a higher body mass index (BMI), male sex, higher glucose, higher low-density lipoprotein (LDL), lower high-density lipoprotein (HDL), increased triglycerides (TG), and higher serum total cholesterol (TC) [2, 4–8]. Currently, no consensus as to treatment has been reached, and tailored, evidence-based treatment for cholesterol polyps is rare. Exploring the risk factors for cholesterol polyp formation, actively controlling the risk factors to reduce the incidence of cholesterol polyps, and decreasing the number of patients undergoing cholecystectomy due to cholesterol polyps are desirable to reduce the surgical trauma and economic costs caused by unnecessary surgery. Because cholesterol polyps are benign lesions, they can be treated without surgery.

Ultrasonography is used for the preliminary diagnosis of GBP, but the final diagnosis requires postoperative pathology. As differences exist in the formation and pathological process between cholesterol GBP and non-cholesterol GBP, a predictor of cholesterol GBP based on pathological classification is more accurate and reliable. However, no such research has been conducted to date. Therefore, this study recruited patients with cholesterol GBP, the dominant type of GBP, according to postoperative pathology from the four-year databases of three high-volume centers, to investigate specific risk factors for cholesterol GBP formation, and to provide a basis for its prevention and treatment.

## Methods

### Patients and design

From January 2016 to December 2019, patients with gallbladder cholesterol polyps that were treated in three hospitals (Department of General Surgery, Xuanwu Hospital, The First Clinical Medical College, Capital Medical University; Department of General Surgery, Peking University First Hospital; Department of General Surgery, Beijing Tiantan Hospital, Capital Medical University) were retrospectively recruited and classified as the cholesterol polyp group. Participants in the non-polyp group were recruited from the health screening center of Xuanwu Hospital. The study protocol was approved by each hospital, and all patients provided informed consent.

For the cholesterol polyp group, the inclusion criteria were as follows: laparoscopic cholecystectomy was performed in patients with gallbladder polyps of minimum size 1.0 cm [9], and the cholesterol polyp group was diagnosed by postoperative pathology. Pathological analysis was independently performed by two senior pathologists and confirmed by a third pathologist. The exclusion criteria were: (1) non-cholesterol polyps or mixed polyps; (2) gallbladder polyps with gallstones; and (3) malignant lesions.

For the non-polyp group, clinical data obtained during annual physical examinations were collected from the database of the Health Screening Center of Xuanwu Hospital, and the results of ultrasound examinations were reviewed. The inclusion criteria were: (1) two or more ultrasonographic examinations; (2) no gallbladder lesions on ultrasonography; and (3) the interval between the first and final examination was longer than 180 days. The results of the final examination were included in the non-polyp group. The exclusion criteria were as follows: (1) presence of gallstones, (2) bile duct obstructive diseases, and (3) acute or chronic cholecystitis. Data from the final follow-up of the non-polyp group were collected and analyzed. The mechanism of the formation of gallstones and cholesterol polyps was unclear, so the confounding factors of gallstones were excluded in both groups.

### Analysis of risk factors

Data characteristics of the cholesterol polyp and non-polyp groups were collected: age, sex, TG, TC, HDL, LDL, systolic blood pressure (SBP), diastolic blood pressure (DBP), alanine aminotransferase (ALT) level, aspartate aminotransferase (AST) level, fasting plasma glucose (FPG), and body mass index (BMI). BMI was calculated by dividing weight (kg) by height squared (m^2^). These laboratory indicators were evaluated routinely. All study participants were subjected to overnight fasting (over 12 h), after which blood samples were drawn from an antecubital vein, and the samples were used for the analysis of biochemical values. Primary data were collected within 24 h after admission in the cholesterol polyp group. For the non-polyp group, the data for each physical examination were recorded in real time. Both groups underwent all the required examinations. Lower HDL was defined as having HDL < 1.04 mmol/L in males and HDL < 1.3 mmol/L in females. Overweight status was defined as a BMI over 25 kg/m^2^, according to the World Health Organization BMI criteria for an Asian population [10].

### Statistical analysis

Statistical analysis was performed using SPSS 21.0 software (SPSS, Inc., Chicago, Illinois, USA). Continuous variables expressed as means ± standard deviation (SD) were evaluated by Student’s t-test, and skewed distribution by Mann-Whitney U test, which are expressed as median (interquartile range). Categorical variables were analyzed using the Chi-square test or Fisher’s exact test. Univariate analysis was performed initially, and variables with statistically significant differences were further analyzed using multivariate logistic regression analysis. *P* < 0.05 was considered statistically significant.

## Results

**Fig. 1:**
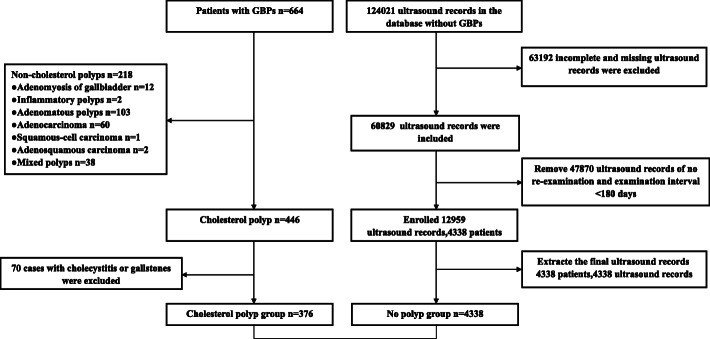
Flow chart of patient screening. Flowchart of inclusion and exclusion in screening of cholesterol polyp group (left) and non-polyp group (right)

This study included a cholesterol polyp group and a non-polyp group. In the cholesterol polyp group, 664 patients with a definite diagnosis of GBP were included through postoperative pathology (Fig. 1). Cholesterol polyps were found in 67.2% (446/664) of the patients. Mixed polyps were defined as the presence of two or more different polyps based on postoperative pathological results, such as cholesterol polyps with adenomyosis, cholesterol polyps with adenomatous polyps, and gallbladder adenomyosis with adenoma. A total of 376 patients with cholesterol polyps confirmed by pathology after cholecystectomy were classified into the cholesterol polyp group after excluding polyps with gallstones or patients with cholecystitis. The mean age of patients in the cholesterol polyp group was 48.28 ± 12.81 years and 41.0% were female.

In the non-polyp group, a total of 124,021 ultrasound results were screened. The study excluded 63,192 cases for which data were not available. Of the remaining 60,829 cases, patients with at least two ultrasound records and a follow-up interval longer than 180 days were selected. The final 4338 patients and their final ultrasound results were included in the non-polyp group. The mean age was 40.9 ± 13.85 years old, and there were 2049 (47.2%) females.

### Univariate analysis of cholesterol polyp formation

Patients in the cholesterol polyp group were older compared with the non-polyp group (48.28 ± 12.81 vs. 40.9 ± 13.85, respectively, *P* < 0.001), fewer were females [154 (41.0%) vs. 2049 (47.2%), *P* = 0.019], had higher BMI (25.04 ± 4.09 vs. 23.88 ± 3.95, *P* < 0.001), higher SBP (125.44 ± 15.67 vs. 120.86 ± 15.99, *P* < 0.001), higher DBP (78.26 ± 10.34 vs. 74.38 ± 10.48, *P* < 0.001), higher LDL (2.91 ± 0.91 vs. 2.81 ± 0.78, *P* = 0.037), lower HDL (1.29 ± 0.75 vs. 1.46 ± 0.37, *P* < 0.001), lower TC [4.30 (1.38) vs. 4.58 (1.14), *P* < 0.001], higher ALT [23.00 (22.00) vs. 17.00 (14.00), *P* < 0.001], higher AST [24.00 (15.00) vs. 21.00 (7.00), *P* < 0.001]. FPG, hypertension, and TG were not significantly different between the two groups (Table 1).

**Table 1:** Univariate analysis of cholesterol polyp group and non-polyp group

	Cholesterol polyp group (*n* = 376)	Non-polyp group (*n* = 4338)	*P* value
Age (years)	48.28 ± 12.81	40.9 ± 13.85	< 0.001^*^
Age > 50 years	240 (63.8%)	1298 (29.9%)	< 0.001^*^
Female sex	154 (41.0%)	2049 (47.2%)	0.019^*^
BMI (kg/m^2^)	25.04 ± 4.09	23.88 ± 3.95	< 0.001^*^
BMI > 25 kg/m^2^	188 (49.9%)	1692 (39.0%)	< 0.001^*^
SBP (mmHg)	125.44 ± 15.67	120.86 ± 15.99	< 0.001^*^
DBP (mmHg)	78.26 ± 10.34	74.38 ± 10.48	< 0.001^*^
Hypertension	68 (18.0%)	737 (17.0%)	0.588
LDL (mmol/L)	2.91 ± 0.91	2.81 ± 0.78	0.037^*^
LDL > 2.89 mmol/L	186 (49.5%)	1822 (42.0%)	0.005^*^
HDL (mmol/L)	1.29 ± 0.75	1.46 ± 0.37	< 0.001^*^
Lower HDL	94 (24.9%)	794 (18.3%)	0.001^*^
FPG (mmol/L)	5.10 (1.13)	5.11 (0.64)	0.971
TC (mmol/L)	4.30 (1.38)	4.58 (1.14)	< 0.001^*^
TC > 5.70 mmol/L	41 (13.0%)	521 (12.0%)	0.526
TG (mmol/L)	1.13 (0.76)	1.08 (0.91)	0.736
ALT (IU/L)	23.00 (22.00)	17.00 (14.00)	< 0.001^*^
ALT > 40 IU/L	68 (18.0%)	521 (12.0%)	0.001^*^
AST (IU/L)	24.00 (15.00)	21.00 (7.00)	< 0.001^*^
AST > 40 IU/L	49 (13.0%)	304 (7.0%)	< 0.001^*^

Values are mean ± standard deviation, n(%) or median (interquartile range)

*BMI* body mass index, *SBP* systolic blood pressure, *DBP* diastolic blood pressure; Hypertension, SBP ≥ 140 mmHg or DBP ≥ 90 mmHg, *LDL* low-density lipoprotein, *HDL* high-density lipoprotein, *FPG* fasting plasma glucose, *TC* total cholesterol, *TG* triglyceride, *ALT* alanine aminotransferase, *AST* aspartate aminotransferase

*P < 0.05

### Logistic regression analysis of cholesterol polyp formation

The comparison between the two groups showed that age > 50 years old [odds ratio (OR) = 3.02, 95% confidence interval (CI) 2.33–3.91, *P* < 0.001], LDL > 2.89 mmol/L (OR = 1.38, 95% CI 1.08–1.78, *P* = 0.011), lower HDL (OR = 1.78, 95% CI 1.32–2.44, *P* < 0.001), AST > 40 IU/L (OR = 3.55, 95% CI 2.07–6.07, *P* < 0.001), and BMI > 25 kg/m^2^ (OR = 1.32, 95% CI 1.01–1.72, *P =* 0.037) were risk factors for cholesterol polyps. Sex, and ALT were not statistically significant factors (Table 2).

**Table 2:** Logistic regression analysis of cholesterol polyp group and non-polyp group

	OR	95% CI	*P* value
Age > 50 years	3.02	2.33–3.91	< 0.001^*^
Female sex	1.23	0.95–1.59	0.126
Lower HDL	1.78	1.32–2.44	< 0.001^*^
LDL > 2.89 mmol/L	1.38	1.08–1.78	0.011^*^
ALT > 40 IU/L	0.94	0.59–1.51	0.808
AST > 40 IU/L	3.55	2.07–6.07	< 0.001^*^
BMI > 25 kg/m^2^	1.32	1.01–1.72	0.037^*^

*HDL* high-density lipoprotein, *Lower HDL* HDL < 1.04 mmol/L in males and HDL < 1.3 mmol/L in females, *LDL* low-density lipoprotein, *TC* total cholesterol, *ALT* alanine aminotransferase, *AST* aspartate aminotransferase, *BMI* body mass index, *OR* odds ratio, *CI* confidence interval

*P < 0.05

## Discussion

Gallbladder polyps are one of the most common diseases worldwide. Cholesterol polyps are the most common form in patients with GBP, and comprised 67.2% in this study. Few previous studies have indicated that cholesterol polyps have the potential to become malignant, and surgical removal of cholesterol polyps may not be necessary. Non-surgical treatment and prevention can bring more benefits to patients by avoiding the trauma and costs associated with unnecessary surgery. Thus, identifying the risk factors for cholesterol polyp formation is important for prevention and individualized treatment. However, previous etiological studies investigating risk factors for gallbladder polyps formation failed to separate cholesterol polyps from other types, such as inflammatory polyps and adenomatous polyps [4, 6, 8]. In addition, false-positive results are inevitable in the diagnosis of cholesterol polyps by abdominal ultrasound, a diagnostic method widely used in previous studies [11, 12]. Thus, this study recruited patients with cholesterol polyps based on pathology to avoid biases from the above confounding factor, in order to determine appropriate preventive as well as interventional measures for cholesterol polyps.

Age was an independent risk factor for the presence of cholesterol polyps. Previous studies have found that the mean age of patients with GBP is 48 years, which is consistent with the results of the study [13, 14]. However, it is worth noting that aging may be related to changes in body metabolism, and previous studies have found that gallbladder polyps may disappear during long-term follow-up [3]. Self-regulation of bile metabolism may affect changes in the gallbladder mucosa. At the same time, in the cholesterol polyp group, a higher proportion of males, which may be related to the influence of female sex hormones on body metabolism. However, further evidence is needed.

LDL could also contribute to cholesterol gallbladder polyp formation, and this is supported by our previous meta-analysis, which did not specify the GBP type [13]. LDL is representative of the status of liver anabolism and cholesterol transport. High LDL can promote the formation of cholesterol polyps by lowering the sensitivity of the gallbladder to cholecystokinin, which subsequently results in decreased gallbladder contraction, cholestasis, and a relative deficiency of cholic acid [15–17]. Meanwhile, the formation of cholesterol polyps may be related to abnormal reverse cholesterol transport related to HDL. These physiological changes can promote cholesterol crystallization and polyp formation. Wu et al. [8] found that TG, TC, HDL, and LDL were not statistically different between the cholesterol polyp group and the non-cholesterol polyp group, indicating that lipid levels may only play a partial role in the formation of cholesterol polyps. Thus, more potential serum indicators are needed to support our hypothesis.

Liver function status has a certain influence on the formation of cholesterol polyps. The specific mechanism of the relationship between the two is unclear, and poor liver function may be related to symptoms of hypermetabolic syndrome, including obesity, hyperglycemia, hyperlipidemia, and hypertension [18]. Lipid metabolism, together with abnormal liver function, may interact closely and simultaneously contribute to the formation of cholesterol polyps. These mechanisms need to be further investigated. Additionally, BMI was an independent risk factor for cholesterol polyps, which is consistent with previous research [7]. Previous studies have found that the formation of GBP is closely related to the overweight status of patients [18–20]. Thus, weight control may help reduce the risk of cholesterol polyp formation. However, epidemiological surveys in western countries have produced different results from this study, which may be related to the differences in diet and living habits of the population [21]. Both liver function metabolism and BMI may be related to abnormal lipid metabolism, which, of course, requires further study.

### Study strengths and limitations

This study is the first to explore the risk factors of cholesterol polyp formation from a pathological perspective, and it was performed based on multicenter data to achieve a better evaluation effect. This study had several limitations. Selection bias may occur in the surgeon’s decision, and a prospective study may be needed in the future. Although serum lipids are closely related to the formation of gallbladder cholesterol polyps and cholesterol calculus, other potential factors, such as bile acid and bile bacteria types, need to be further studied to illustrate the specific mechanism of cholesterol polyp formation [22].

## Conclusion

In conclusion, this study aimed to analyze the risk factors for cholesterol polyps on a pathological basis. Age, LDL level, HDL level, AST level, and BMI were independent risk factors for cholesterol polyps. Elderly patients should control their weight, protect the liver, and adjust their blood lipid levels, which may help reduce the incidence of cholesterol polyps. Therefore, further prospective studies are required.

## Data Availability

The data in this article is dependable. The original data is available from the corresponding authors.

## Abbreviations

BMI: Body mass index
SBP: Systolic blood pressure
DBP: Diastolic blood pressure
LDL: Low-density lipoprotein
HDL: High-density lipoprotein
TG: Triglycerides
TC: Total cholesterol
AST: Aspartate aminotransferase
ALT: Alanine aminotransferase
GBP: Gallbladder polyps
FPG: Fasting plasma glucose
OR: Odds ratio
CI: Confidence interval
SD: Standard deviation
